# Machine Learning Applications in the Neuro ICU: A Solution to Big Data Mayhem?

**DOI:** 10.3389/fneur.2020.554633

**Published:** 2020-10-09

**Authors:** Farhan Chaudhry, Rachel J. Hunt, Prashant Hariharan, Sharath Kumar Anand, Surya Sanjay, Ellen E. Kjoller, Connor M. Bartlett, Kipp W. Johnson, Phillip D. Levy, Houtan Noushmehr, Ian Y. Lee

**Affiliations:** ^1^Department of Emergency Medicine and Integrative Biosciences Center, Wayne State University, Detroit, MI, United States; ^2^Department of Neurosurgery, Henry Ford Hospital, Detroit, MI, United States; ^3^Department of Biomedical Engineering, Wayne State University, Detroit, MI, United States; ^4^Department of Genetics and Genomic Sciences, Icahn School of Medicine at Mount Sinai, New York, NY, United States

**Keywords:** neurocritical care, machine learning, artificial intelligence, neurology, intensive and critical care

## Abstract

The neurological ICU (neuro ICU) often suffers from significant limitations due to scarce resource availability for their neurocritical care patients. Neuro ICU patients require frequent neurological evaluations, continuous monitoring of various physiological parameters, frequent imaging, and routine lab testing. This amasses large amounts of data specific to each patient. Neuro ICU teams are often overburdened by the resulting complexity of data for each patient. Machine Learning algorithms (ML), are uniquely capable of interpreting high-dimensional datasets that are too difficult for humans to comprehend. Therefore, the application of ML in the neuro ICU could alleviate the burden of analyzing big datasets for each patient. This review serves to (1) briefly summarize ML and compare the different types of MLs, (2) review recent ML applications to improve neuro ICU management and (3) describe the future implications of ML to neuro ICU management.

Care-provider teams in the neurological intensive care units (neuro ICUs) routinely interpret large and heterogeneous patient datasets including data types such as physiological waveforms, continuous electroencephalograms, laboratory tests, and images (1). This has proven to be extensively difficult and time consuming, and may sometimes result in ICU providers being unable to incorporate critical information into clinical decision-making in a way which may impact patient outcomes. Thus, there is a significant clinical need to aid clinical decision-making by automatically interpreting large sums of data collected in the neuro ICU to both reduce resource utilization and improve patient care.

Machine learning (ML) is a subfield of artificial intelligence (AI) focused upon the creation of algorithms to model large data sets and make predictions. The ability of ML to use large, heterogeneous data sets to discover previously unknown patterns and associations has led to improved prediction accuracy of patient outcomes in the ICU (2). Therefore, ML has gained tremendous recognition as a potential solution to the neuro ICUs' large data problems by rapidly interpreting data to assist in clinical decision-making (Figure 1).

**Figure 1:**
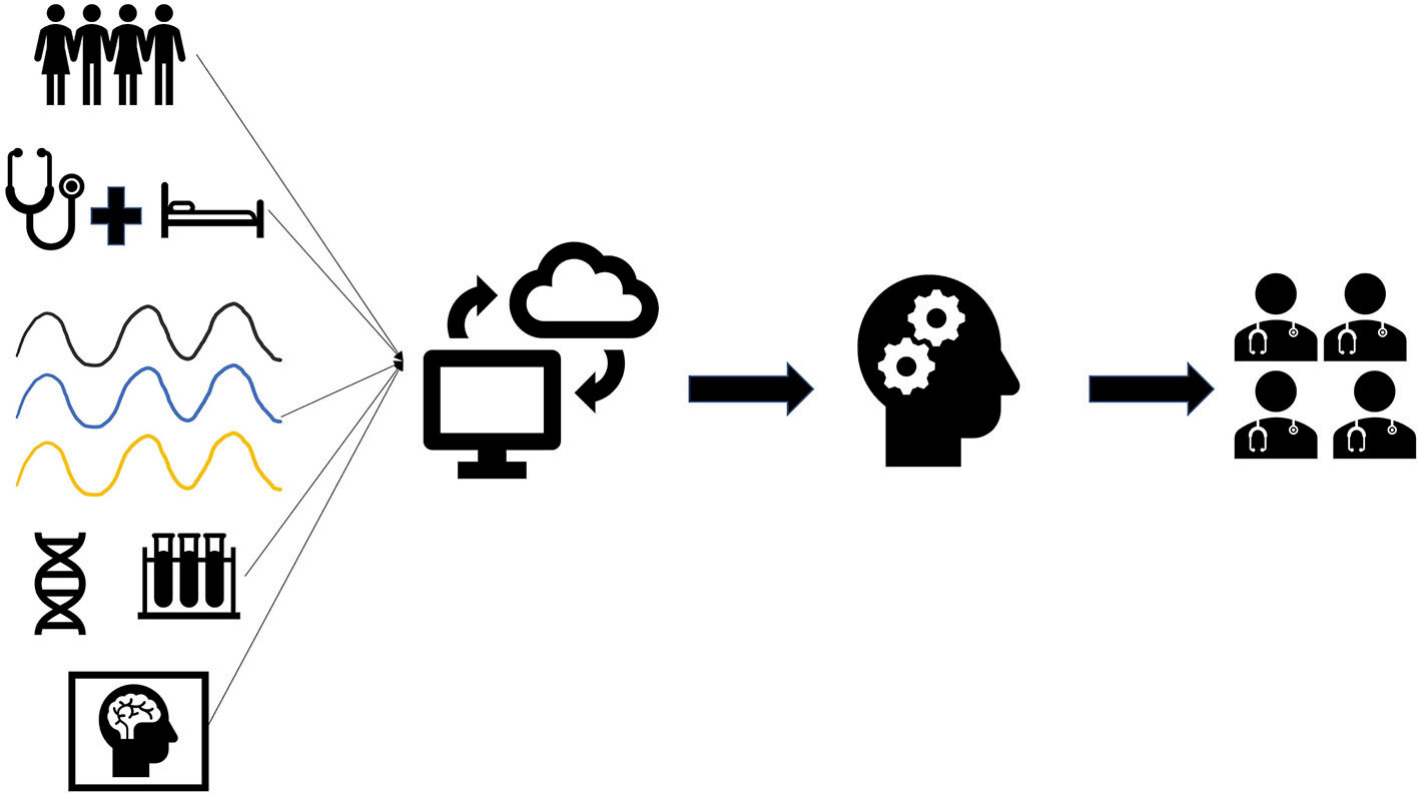
Overview of data collection and implementation for machine learning. Neuro ICU data including patient demographics, clinical findings, waveforms, genetics, lab results, and imaging are collected in the electronic medical records. Data collected from patients can be uploaded onto a universal data cloud for research purposes. Likewise, large datasets from other neuro ICUs can be downloaded for analysis. Machine learning (ML) will then be trained using patient data collected from the neuro ICU and/or cloud. ML can then be used by neuro ICU teams to assist in clinical decision-making for their patients.

The major hurdles when utilizing MLs in clinical practice are often the lack of awareness and comfort physicians have with MLs (3). Therefore, our overarching aim is to increase awareness of how ML may benefit neuro ICU physicians to help them discover the potential of MLs in their clinical practice. There have been several reviews summarizing the role of ML in neurology/neurosurgery (4–6). This review will (1) briefly summarize ML and compare the different types of ML commonly used in neuro ICU research, (2) specifically review recent ML applications to improve neuro ICU management and (3) describe the future implications of ML to neuro ICU management.

## Machine Learning vs. Standard Statistical Approaches

“*Medicine is a science of uncertainty and an art of probability” - Sir William Osler* (7).

The first and most important distinction for clinicians to understand is the difference between ML and standard statistical approaches (SSA), such as linear and logistic regression.

### Fundamentals of Scientific Inquiry

To better understand how this difference is pertinent to clinicians, one must be reminded of the fundamentals of scientific inquiry. We gain knowledge by making inferences based on results obtained from testing a hypothesis via scientific experimentation. This knowledge is then used to make predictions, ultimately influencing clinical decision-making. An example of an inference is that patients with higher blood pressures suffer more stroke events when compared to those with lower blood pressure. Therefore, we can make inferences to discover the exact relationship between stroke and blood pressure–does it increase monotonically, what is the degree of increased risk per increment of blood pressure, how does this relationship change in the context of other variables, etc. In contrast, ML is focuses more on accurate prediction alone (at least in the case of supervised learning). In actuality, statistics and machine learning overlap and are complementary. Referring to Sir Osler's famous quote, there is often a significant degree of uncertainty in medicine, thereby making an accurate prediction an artistic process based on evidence-based knowledge from the literature and clinical know-how (7). These two broadened steps of first making an inference from clinical studies and then making predictions, albeit related, are often studied independently from one another. For example: we can identify statistically meaningful differences or relationships between variables using SSAs; however, this may not have any substantial impact on the prediction of an outcome. Conversely, we can make accurate predictions of outcomes, without understanding the relationships and interactions of the different variables on the outcome.

### Complexities of Datasets

As clinicians, one may argue that we would care more about which clinical decision-model results in improved outcomes for our patients, rather than why would these decisions improve their outcomes. Often, as mentioned before, the number of variable interactions (or dimensions) within healthcare data (i.e., age, sex, blood pressure, past medical history, thousands of potential drugs or procedures, imaging results composed of pixels with higher order structures, millions of genetic polymorphisms, etc.) is so high that it becomes troublesome to identify relationships with traditional statistical models (8). Therefore, it has proven to be difficult with SSAs to make accurate predictions or statistically significant inferences on multi-dimensional datasets with potentially numerous non-linear relationships (8).

MLs, on the other hand, have demonstrated better predictive capabilities, for larger more multi-dimensional datasets (9). ML models can identify non-linear patterns by making fewer assumptions within the data (e.g., statistical relationship) resulting in the best predictive performance. Consequently, due to this non-linear nature, MLs may fail to provide statistically meaningful interactions within the dataset. For example, even the less complicated random forests algorithms can be used to probabilistically model outcomes in a non-linear fashion, however they are less useful for the objectives of statistical inference. Therefore, we understand what the inputs and outputs are for the algorithm, but often do not understand the internal workings of the model (aka “black box model”) (10). Thus, we often ignore the “why” in the relationship of certain variables in these models to improve predictive power by sacrificing interpretability of the model (10).

### Predictiveness vs. Interpretability

**Figure 2:**
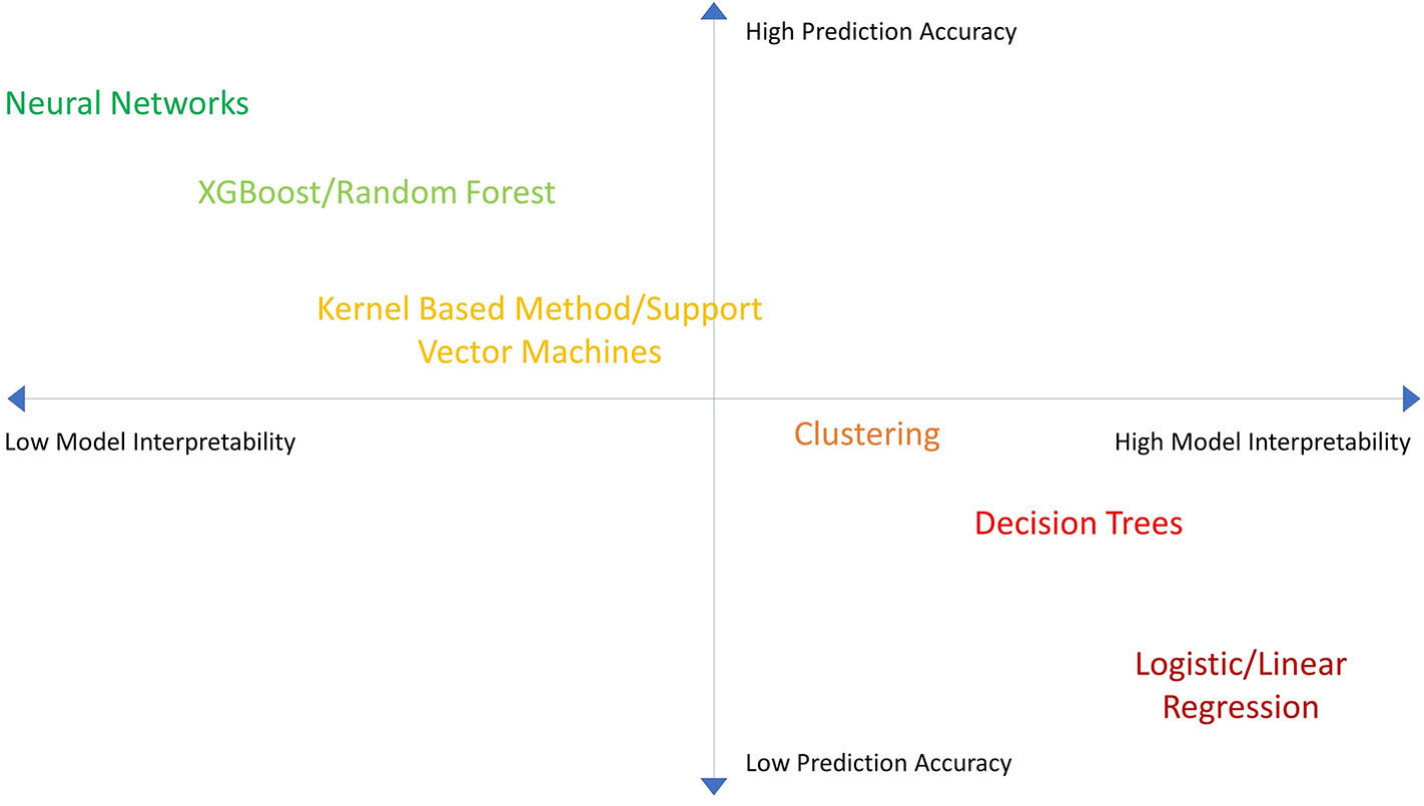
Machine Learning model accuracy vs. interpretability. A chart showing the performance of different machine learning (ML) algorithms in terms of model accuracy vs. interpretability. MLs are plotted in relation to one another in terms of performance.

MLs have trade-offs between predictiveness and interpretability based on the complexity of the model (Figure 2). Additionally MLs have variable utility based on the dataset. For example: artificial neural networks (ANN) tends to lose predictive power with smaller datasets (11). Thus, we must weigh the relative pros and cons of different MLs and linear-SSAs to determine what analytic approach would be most suitable to reach our goal (9–11) (Table 1). Most importantly, since MLs make different assumptions regarding the datasets they train on, some assumptions may not hold true for every dataset. Consequently, one ML model may not work best for every dataset (12). Therefore, one must try multiple ML and SSA models for each dataset to identify the “best” model.

**Table 1:** Comparison of Machine learning approaches vs. standard statistical approaches.

	**Machine learning**	**Standard statistics (linear/logistic regressions)**
Data preparation?	Doesn't require explicit commands to find patterns in data	Need to know variables and parameters beforehand
Hypothesis?	No hypothesis needed	Need hypothesis to test
Type of data?	Multi-dimensional data that can be non-linear in nature	Linear data
Training?	Needs to be “Trained”	No training
Goal?	Generally better for predictions	Generally better for inferences/hypothesis testing
Scientific question?	What will happen?	How/why it happens?

*Table compares the differences between machine learning (ML) and standard statistical approaches (SSA). These comparisons are simplified rules, and therefore may not hold true for every dataset as size and quality of dataset can alter the performances of MLs and SSAs*.

In general, nonetheless, MLs tend to have better predictive strength while SSAs tend to have better interpretability. Therefore, the differences between MLs and SSAs are conceptually analogous to the differences between forks and spoons. Both tools are important, however one tool will tend to be superior to the other based on what the intended use is.

## The Machine Learning Approach

Overall, there are three main tasks for ML: (1) regression, (2) classification, and (3) clustering.

### Regression

Regression is the ability to model and predict continuous, numeric variables (i.e., age, blood pressure number, intracranial pressure number etc.). It is a supervised learning model whereby the algorithm is trained and then tested on a labeled dataset (i.e., desired outcomes for patients have been identified and labeled) (13). A traditional SSA approach to this would be linear regression analysis which will attempt to find and quantify a statistically significant linear relationship between two variables. If there are multiple independent variables involved in the regression model, a multivariable regression analysis can be used to find statistically significant relationships amongst the variables. It has been shown that as the amount of independent variables increases, and thus the dataset becomes more multidimensional, ML-based methods such as support vector machine regression or tree regression models are more robust in making predictions for these types of datasets when compared to SSA (3, 14).

### Classification

Classification is another supervised learning model that is used to model and predict categorical variables (does this EEG show a seizure or not?, is there evidence of a intracranial bleed or not? etc.). The predictive strength of a classifying model is often represented by a receiver operating characteristic curve (ROC curve) where the area under the curve (AUC) summarizes the overall performance of the discriminative model (15). However, for unbalanced datasets where a specific outcome may be rare (i.e., out of 100,000 patients, only 100 died) one should instead consider a precision-recall curve (PRC) to assess the strength of a classifying algorithms. PRCs examine the trade-off between true positive rates and positive predictive value and are useful in assessing the strength of algorithms which predict rarer outcomes (16).

In SSA, we often use logistic regression or multivariate logistic analysis which is like their regression counterparts except that it is used to make categorical regressions. Similarly, it has been shown that for higher dimensional datasets that more advanced ML classification methods can outperform these SSA methods by identifying non-linear patterns to better predict a categorical outcome (14).

### Clustering

Clustering is an unsupervised learning technique that finds groupings of observations in a dataset without reference to external labels. This method creates clusters of data points with similar properties and features. It is unsupervised because there are no prior labeled categories (e.g., we do not know the survival outcomes for the patients), and the algorithm will therefore create its own categories. The main goal of clustering is to identify new groups without bias within a dataset. For example, we can automatically group patients based on similar clinical characteristics that they share without knowing anything about their respective outcomes. Clustering is also useful in imaging analysis as it can automatically group and differentiate images based on its appearance.

## Types of Machine Learning

Several different review papers have extensively written summaries of the different types of MLs. Therefore, we will focus on describing, briefly, the most used MLs in neuro ICU research. Commonly used MLs in ICU research, in general, include neural networks (42.6%), support vector machines (23.7%), and classification/decision trees (20.1%) (2).

### Support Vector Machines

Support vector machines (SVMs) essentially allow us to map our input data in a non-linear fashion, which is then separated into classes that are thus non-linear. SVMs are known to be robust to overfitting and work well with high-dimensional datasets; however, they have been shown to scale poorly to larger datasets and datasets with excessive noise where the outcome-categories overlap (13).

### Classification/Decision Trees

Trees learn in a hierarchical fashion where the dataset is repeatedly divided into separate branches. This forms a flowchart structure where each split is a test on a predictor variable (high blood pressure). A branch is formed that represents whether the variable was present or not (i.e., “yes” or “no”). An accretion of these branches forms a tree that accumulates to make a decision-rule regarding a specific outcome. Ensemble methods such as random forest and gradient boosted trees (i.e., XGBoost) combine predictions from many different decision trees to identify the optimal decision pattern. In each permutation of trees, different variables are given different values of importance. Therefore, you can identify the most important variables in the model. Decision trees can remain robust in the presence of outliers and are able to interpret non-linear relationships effectively. Ensemble methods are believed to be the best MLs for smaller to medium-sized structured data sets (17, 18).

### Neural Networks

The premise of neural network MLs is based on the human neural network in the brain. Neural networks train on datasets with labeled variables and outcomes (supervised learning) to then cluster the data (unsupervised learning), identifying new patterns related to an outcome. Deep learning is when there is a multistep process for pattern recognition utilizing layers of neural networks. Each layer builds on one another, where the output of the first layer must be interpreted by the next layer and so on. This allows the deep-learning network to train on features detected from the previous layer to improve the automatic abstraction of important patterns in a dataset. This is most likely the reason why deep learning method are the most frequently studied MLs for ICU datasets (2, 13). They can automatically extract features from formats that are often difficult to extract without bias. For example, before deep learning, imaging and waveform data classification tasks often required hand-tuned extraction or labeling of “features” by trained data collectors (i.e., a researcher would classify a type or severity of an intracranial bleed based on training or pre-existing guidelines). Deep learning can automatically extract features from images or waveforms and input these features into the algorithm to come up with an unbiased classification scheme. Unsurprisingly, for electroencephalograms (EEGs), deep learning methods have been extensively used to improve EEG-interpretation (5, 19).

A significant limitation to most deep learning methods is that they often require very large amounts of data to outperform tree based MLs; though, there are some artificial neural networks that have been studied for smaller datasets (20). Furthermore, increasing computational complexity of deep learning algorithms by adding more nested layers between input and output make these algorithms more difficult to reproduce experimentally (10, 19).

## Search Strategy and Selection Criteria

References for this Review were identified by searches of PubMed between 1970 and January 2020. The search terms “Machine Learning,” “Artificial Intelligence,” “Neurocritical care,” “Neurological intensive care unit,” and “Neuro ICU” were used. There were no language restrictions. The final reference list was generated based on strength of study and relevance to the topics covered in this Review.

## Machine Learning to Improve the Assessment of Consciousness in the Neuro ICU

Assessment of consciousness via clinical examination is an essential aspect in the management for neuro ICU patients, however, this assessment has proven to be challenging (21–23). Additionally, level of consciousness frequently waxes and wanes, making an accurate assessment even more difficult.

### EEG and Imaging of Consciousness

Current advancements in functional magnetic resonance imaging (fMRI), and EEGs, have resulted in a more objective assessment of level of consciousness (22). Some patients at bedside may not show signs of consciousness when commanded to move a muscle; however, they may be able to show modulation of brain activity, when assessed by fMRI or EEG, in response to that command (24). This occurrence known as “cognitive-motor dissociation” is believed to be an activation of cerebral neural circuits in response to command, but producing no motor or verbal response. Amazingly, this phenomenon is believed to occur in 14% of chronically unresponsive patients (22).

The clinical importance of cognitive-motor dissociation still needs to be determined, and therefore it warrants larger scale multi-center trials. Furthermore, its assessment requires more efficient monitoring of continuous EEGs and fMRIs in unresponsive patients in the neuro ICU. Claassen et al. (25) utilized an SVM to rapidly read EEGs from unresponsive and healthy patient to detect cognitive-motor dissociation. MLs, such as SVMs, have been shown to efficiently interpret large-scale datasets of EEGs. The SVM was trained and then prospectively tested on 240 EEG readings after given the command to move the hands. Although the SVM had variable EEG classification success for cognitive response (i.e., AUC ranged from 0·40–0·75 depending on underlying pathology), the study did show an increased likelihood for neurological recovery for unresponsive patients whom the SVM detected a cognitive response on the EEG (OR: 4·6; 95% CI: 1·2–17·1).

It should be noted, however, that the etiology leading to unresponsiveness significantly varied from patient to patient, resulting in more classification noise thereby hindering the SVM's performance. For future trials, more sophisticated MLs such as ANNs maybe better adept to handing the complex nature of these datasets, though this would most likely require a much larger sample size of patients. Nonetheless, the utilization of ML proved to be an efficient modality to interpret these long continuous EEG readings in the neuro ICU, leading to improved prognosis prediction.

## Machine Learning Improves Monitoring With Intracranial Pressure

Continuous intracranial pressure (ICP) monitoring is a routine practice in neuro ICUs in attempts to identify increases in ICP associated with decreased cerebral perfusion (26). The current gold standard measurement for ICP is by intraventricular catheters. Non-invasive ICP measures have been studied, however, as of now, have not produced significant clinical success, and are therefore not routinely used (26).

Raj et al. (27) developed a dynamic multiple regression prediction model to predict 30-day all-cause mortality after traumatic brain injury (TBI). The model used variables gathered from continuous ICP waveform measurements from intraventricular catheter or an intraparenchymal probe and other physiological measurements (i.e., mean arterial blood pressure). The dynamic model showed good predictive strength for mortality after initial presentation (highest-AUC = 0·84) in comparison to the IMPACT-TBI score (highest-AUC = 0·78). Although the overall performance of the dynamic model was only modestly better, it allowed for more individualized readings by incorporating rapid real time predictions for each patient. Therefore, adding continuous waveform monitoring could significantly improve prognostic accuracy and precision for predictive ML models.

## Machine Learning Approaches to Monitor Intracranial Hemorrhage

### CT Triaging

Automation of reading CTs could improve the triaging of suspected intracranial bleeds, thereby reducing potentially harmful delays in stat readings by overburdened radiology departments. Arbabshirani et al. (28) trained a convolutional neural network (CNN) on 37,074 images from different facilities and tested it prospectively for 3 months to better prioritize suspected ICHs for radiologists in real time. This predictive model showed good classification of ICH (AUC = 0·846) when compared to radiologic reports. The CNN successfully reprioritized 60 patients from “routine” to “stat” read for suspected ICH. Amazingly, the CNN was also able to identify five missed cases of ICH while reducing the median time to diagnosis from 512 to 19 min. Even though outcome data was not recorded, it can reasonably be hypothesized that improving time to diagnosis and improving detection rates of small ICHs via computer-aided-CT could improve outcomes while reducing workload.

### Biomarkers for Prediction of Delayed Cerebral Ischemia

Delayed cerebral ischemia (DCI) is a common complication following subarachnoid hemorrhages in the Neuro ICU. Extracellular proteins (ECPS), such as osteopontin, periostin, and galactin-3, are believed to be involved with inflammation, angiogenesis, fibrosis, vasogenic permeability and cellular death mechanisms; therefore, these ECPs could be clinical biomarkers for DCI demonstrating significant prognostic value (29–31).

Tanioka et al. (31) attempted to construct a random forest prediction model that incorporated these ECPs to better predict DCI and angiographic vasospasm after subarachnoid hemorrhage. They tested three different models: model 1 only included clinical variables on admission, model 2 only included plasma ECP levels 1–3 days after initial presentation, and model 3 incorporated clinical variables and plasma ECP levels 1–3 days after admission. They studied these models in a prospective cohort of 95 newly diagnosed patients with subarachnoid hemorrhage. The prediction accuracy of model 3 (mean value = 95·1%) was modestly superior to model 1 (mean value = 93.9%), but significantly superior to model 2 (mean value = 87·2%) for predicting clinically-diagnosed DCI (neurological deterioration clinically-assessed at bed side). This study clearly demonstrated that MLs can integrate a multitude of multidimensional datasets to improve their predictive performance.

## Machine Learning to Improve Detection of Seizures in the Neuro ICU

Continuous EEG (cEEG) monitoring is the gold standard to detect subclinical seizures in the neuro ICU; however, cEEG reading is time-consuming and labor-intensive resulting in a significant limitation of its use (32). Despite the fact that cEEG reading-automation with MLs have been extensively studied in seizure detection, their application into the Neuro ICU, so far, have been limited due to their complex nature. The development of user-friendly ML-based software could promote the integration of automated EEG technologies into the neuro ICU.

To better monitor seizures in the Efficacy of Intravenous Levetiracetam in Neonatal Seizures trial (NEOLEV2), the company Persyst developed an automated software to analyze cEEGs of participants (33). The software provided real-time detection of seizure activity leading to quicker detection rate, though, it failed to reduce workload for neurologists due to its subpar accuracy, therefore requiring human review. RiskSLIM is a sparse linear integer machine that showed good predictive accuracy (AUC = 0·83), comparable to other commonly used MLs (34). Additionally, Koren et al. (35) showed that the ML-based software called Neurotrend, had good detection accuracy for certain seizure activity while reducing time for cEEG review; however, inter-agreement between users was poor for rhythmic-periodic waves and unequivocal EEG patterns. Software that use MLs to monitor seizure activities in the Neuro ICU are currently in-high demand, and we expect that there will be more commercially available soon.

## Machine Learning to Better Predict Hemorrhagic Transformation

Hemorrhagic transformation (HT) is a rare, yet, serious complication following thrombolytic therapy for stroke (36). There are many interrelated risk factors that are associated with HT; thus, making it difficult to predict its risk for each patient. Since MLs are adept to incorporate multiple patient variables to predict an outcome, MLs may be promising tools to improve the prediction of HT.

Yu et al. (37) compared multiple ML algorithms that used source perfusion MRI information and patient information (age, NIHSS, diabetes, baseline serum glucose) to predict severity and location of HT in patients with acute stroke. The ML automatically extracted features from perfusion-weighted imaging MRI (PWI) before thrombolytic therapy in attempts to predict HT, assessed by gradient recalled echo angiography taken 24 h after therapy. One hundred and fifty five stroke-patients were analyzed. Forty one of these patients would eventually have HT. The Kernel Spectral Regression for Discriminant Analysis (SR-KDA) ML model accurately predicted hemorrhagic transformation in 88% of patients (AUC = 0·84), outperforming linear regression (AUC = 0·58), decision-tree models (AUC = 0·80), SVM (AUC = 0·82), and neural network (AUC = 0·81).

## Machine Learning to Predict and Diagnose Nosocomial CNS Infection in the NEURO ICU

Neuro ICU patients are uniquely at risk for acquired CNS infections associated with invasive neurosurgical procedures. Nosocomial CNS infections result in increased length of stay and increased mortality in neuro ICU patients. Mitigation of risk factors, early detection, and prompt treatment is therefore critical (38).

Savin et al. (39) studied two groups of high-risk Neuro ICU patients, one group with hospital-acquired ventriculitis and meningitis (HAVM, *n* = 216) and one group without HAVM (*n* = 2,070) (39). They identified with the advanced ensemble method, XGBoost, the four most important risk factors involved with HAVM: (1) presence of external ventricular device, (2) recent craniotomy, (3) presence of superficial surgical-site infection, and (4) CSF leaks. Furthermore, they identified that increasing the number of days of EVD significantly increases the predictive-probability for HAVM, thus supporting previous findings showing that the number of days of EVD placement proportionally correlates with an increased risk of HAVM (40). It is therefore suggested that clinical prevention of HAVM should focus on these four risk-factors. Future randomized clinical trials are warranted, but it can be expected that limiting invasive devices and removing invasive devices as soon as possible would substantially reduce HAVM-risk.

## Machine Learning to Determine Neurological Recovery After Neuro ICU Stay

Prognostic accuracy has significant medical and ethical implications on patient care. Better prognosis offers reasonable hope and often results in more aggressive management, while poorer prognosis usually results in comfort-of-care (41). Therefore, prognostic accuracy needs to be correct and evidenced-based. Considering various factors affecting prognosis, MLs may aid physicians to make a more precise and unbiased prognosis prediction.

Stapleton et al. (42) utilized elastic net with LASSO to analyze over 150 metabolites obtained via mass spectrometry in 137 patients with subarachnoid hemorrhages; the goal was to identify metabolites that were most predictive of 90-day functional outcomes (determined by a modified Rankin Scale) (42). They found that increased plasma taurine, which is a non-neurotransmitter amino acid that is found abundantly in human brains, is independently associated with functional outcomes at 90 days post-subarachnoid hemorrhage. It is known that taurine attenuates inflammation and oxidative stress and has been shown to modulate neuronal activity (43). Therefore, monitoring plasma taurine levels in conjunction with other circulating biomarkers could provide significant prognostic information following subarachnoid hemorrhage.

Hernandes Rocha et al. (44) trained several ML algorithms on a large TBI registry in a remote hospital in Moshi, Tanzania where there are no ICP monitoring devices and limited access to neurosurgical services. They were able to find that the Bayesian generalized linear model had good predictive accuracy (AUC = 0·865) for determining the neurological function at discharge for newly admitted TBIs. The ML incorporated predictors that were practical for low-resource facilities to obtain, including age, sex, and knowledge of history of present illness, alcohol use, vitals, and glucose levels. MLs that use readily available prognostic markers could significantly improve decision-making outcomes for TBI patients in resource-depleted areas by integrating multiple readily available factors.

## Machine Learning Applications in Hydrocephalus for Neonates

Neonatal hydrocephalus is a common concern seen in the neonatal ICU. Most cases of hydrocephalus are progressive resulting in significant neurological deterioration if not managed effectively (45). Therefore, improving clinicians' ability to predict hydrocephalus earlier is crucial to improve outcomes. Furthermore, since neonatal-neuro ICUs and pediatric neurosurgeons are significantly limited resources, early prediction of potentially high-risk neonates during prenatal assessment could significantly improve planning for the neonate after delivery.

Tabrizi el al. (46) retrospectively studied premature neonates and extracted morphological features of the lateral ventricles from cranial ultrasound (CUS) imaging to predict the outcome of post-hemorrhagic hydrocephalus (PHH), secondary to intraventricular hemorrhage (IVH) (46). Their model utilized an SVM and was able to predict the need for intervention for PHH with a high accuracy of 84%.

Heaphy-Henault et al. (47) also conducted a retrospective study to identify morphological features of cerebral ventricles observed on fetal MRI images that could forecast the likelihood of post-natal congenital aqueductal stenosis (CAS). They extracted features related to CAS, obstructive hydrocephalus and associated malformations from pre- and post-natal brain MR images. This approach allowed them to estimate the independent contributions of individual variables (such as enlargement of the inferior recesses of the third ventricle, size of the lateral and third ventricles and an abnormally thin and/or dysgenetic corpus callosum) while accounting for the contributions of other variables as well. Their model was able to identify enlargement of the inferior recesses of the third ventricle as the most predictive findings for post-delivery CAS.

Pisapia et al. (48) applied an SVM to investigate cerebral ventriculomegaly to better predict the need for shunt placement from a fetal MRI. They attempted to detect patterns of features in images that were not appreciable by visual inspection alone. The algorithm was able to correctly classify post-natal CSF diversion status with 82% accuracy. The authors then ran the SVM on an independent replication cohort study where the model achieved 91% accuracy. We anticipate similar studies in the future showing MLs that could provide significant advantages for neurosurgeons by allowing them to better assess neonates before birth.

## Limitations

With the growth of computational power and the increase in data availability and monitoring, MLs are becoming an exciting tool in neuro ICU research. Nevertheless, its application to clinical practice has been limited. One of the main limitations to the application of MLs in clinical practice is the ethics behind relying on MLs for clinical decision-making. Even though MLs have been shown to outperform standard protocols for clinical decision-making, there is a significant concern regarding who is liable if an ML makes an error. Furthermore, as explained before, due to the “black box” nature of MLs, it would be difficult to identify the source of error if made by an ML. Understandably; not comprehending the process of how an ML makes a conclusion can lead to significant hesitancy when applying MLs to the clinical decision-making process (10). Therefore, MLs, currently, should be used primarily to facilitate decision-making, like that seen with current risk-scoring systems.

**Figure 3:**
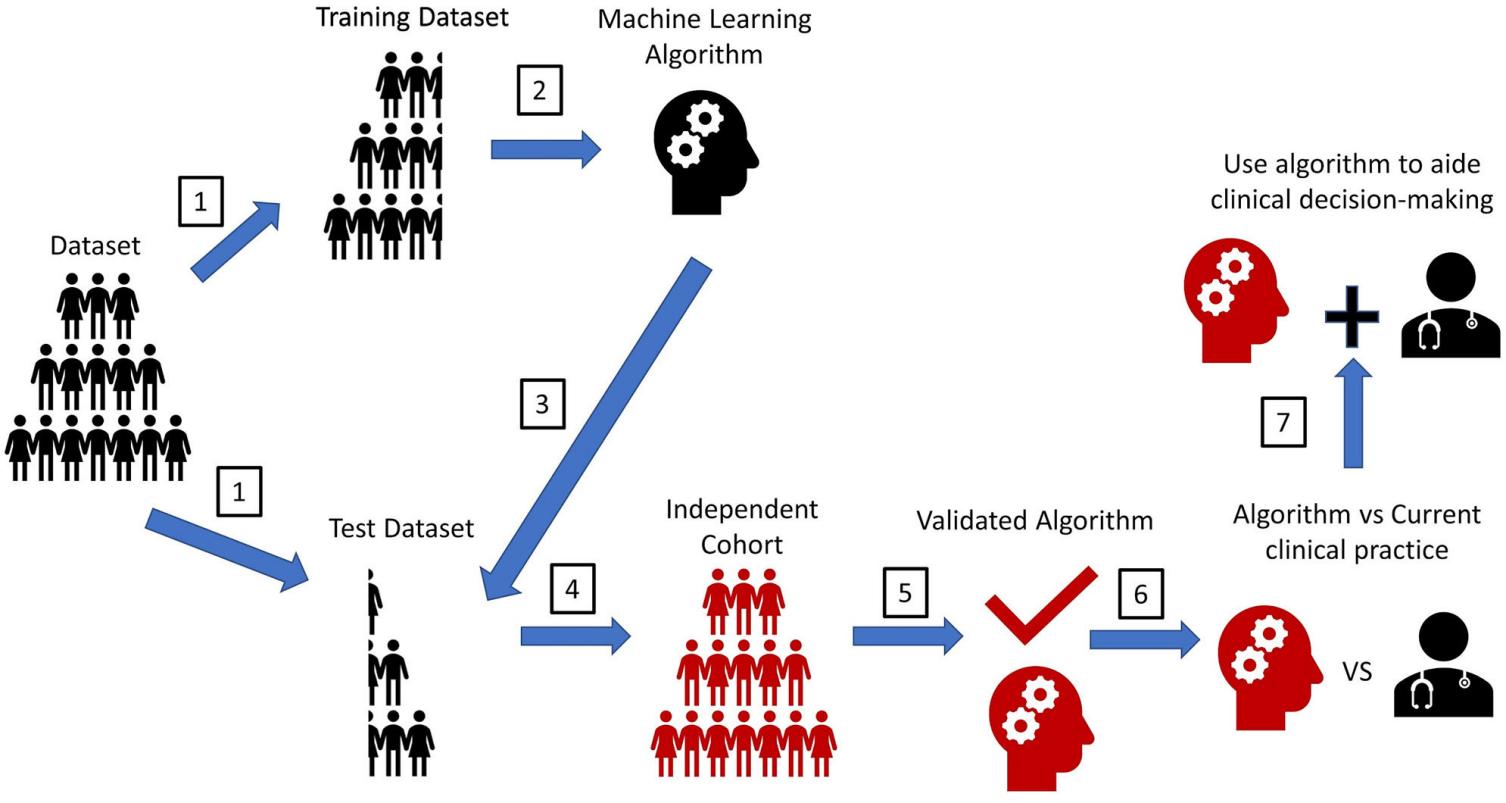
Experimental workflow for machine learning algorithms. A pipeline diagram for the ideal machine learning (ML) experiment. This pipeline is simplified into seven steps. (1) Retrospectively collected datasets with labeled outcomes need to be randomly split into a training set and a test set. The method to split the dataset to create a training dataset and test dataset vary based on the type cross-validation method. (2) ML will train on the training set to make predictions for the labeled outcome. (3) ML predictions of an outcome will be compared with the actual outcomes in the test set to determine prediction accuracy. (4) The gold standard assessing ML performance is to test the ML prediction accuracy on another dataset that is completely independent from the original training dataset. (5) This step will validate the performance strength of the ML. (6) To assess the MLs clinical utility, a prospective trial should be conducted. The ML's prediction accuracy of an outcome should be compared with that seen from the current clinical standard-of-care (i.e., current scoring system, physician prediction or diagnosis). If prediction accuracy of the ML is non-inferior or greater than that seen from the current clinical standard-of-care, the ML should be used to assist in the clinical decision-making. Ideally, the ML would be implemented in the electronic health record (EHR) to automatically extract pertinent patient data for each patient to aid decision-making, like how certain risk-stratification scores are automatically calculated in some EHR systems. This will help reduce workload burden for clinical teams, while also possibly improving the accuracy of clinical decision-making. Furthermore, since MLs have an automatic process for interpreting patient data, MLs could help teams reduce any potential biased-based decisions made by different rounding ICU teams.

Other significant limitations to ML applications in clinical research include accumulating and analyzing large datasets for prospective studies to validate ML-based models. Majority of ML studies mentioned previously trained and tested the MLs on retrospective data only (Table 2). The gold standard to validate MLs is to test the ML on an independent cohort prospectively, yet, only a minority of the previously-mentioned studies validated their ML on an independent cohort (Figure 3). Furthermore, studies that utilized independent cohorts were often small and were not from multiple centers.

**Table 2:** Summary of studies mentioned in review.

**Publication**	**Indication**	**Type of machine learning algorithm**	**Size of dataset**	**Performance of model tested**	**Tested on prospective dataset**
Claassen et al. (25)	Detection of cognitive-motor dissociation on EEG	SVM	240 EEGs from 104 patients	Significantly improved prognosis of neurological recovery (OR 4.6; CI 1.2–17.1)	Yes
Raj et al. (27)	Predict 30-day all-cause mortality after TBI with invasive ICP measurements	Modified logistic regression using dynamic variables	472 patients	AUC = 0.84	No
Arbabshirani et al. (28)	Detection of intracranial hemorrhage	CNN	46,583 CT scans	AUC = 0.85	Yes
Tanioka et al. (31)	Prediction of DCI after subarachnoid hemorrhage	Random Forest	95 patients	Prediction accuracy = 95.1%	Yes
Struck et al. (34)	Seizure prediction using cEEGs	RiskSLIM	7,716 cEEGs	AUC = 0.83	Yes
Koren et al. (35)	Assist EEG reviewers to annotate different cEEG patterns	Neurotrend	76 cEEGs	Multi-rater agreement for burst suppression (Gwet's coefficient = 0.86)	No
Yu et al. (37)	Predict hemorrhagic transformation	Kernel spectral regression	165 patients	AUC = 0.84	No
Savin et al. (39)	Prediction of healthcare-associated ventriculitis and meningitis	XGBoost	2,286 patients	AUC = 0.83	No
Stapleton et al. (42)	Identification of metabolite associated with neurological outcomes following subarachnoid hemorrhage	LASSO Regression	137 patients	Found plasma levels of taurine were 21.9% higher in patients with good vs. poor outcomes (*P* = 0.002)	No
Hernandes Rocha et al. (44)	Predict neurological recovery following TBI	Bayesian generalized linear model	3,138 patients	AUC = 0.87	No
Tabrizi et al. (46)	Predict post-hemorrhagic hydrocephalus outcomes in neonates with intraventricular hemorrhage using cranial ultrasound	SVM	64 patients	Prediction Accuracy = 84%	No
Heaphy-Henault et al. (47)	Predict congenital aqueductal stenosis with fetal MRI and the most important fetal MRI findings associated with congenital aqueductal stenosis	Random Forest	75 patients	Found enlarged inferior recesses of the third ventricle were the most important fetal MRI features associated with congenital aqueductal stenosis (*P* < 0.0023)	No
Pisapia et al. (48)	Predicting which patients would require post-natal cerebrospinal fluid diversion with fetal MRI	SVM	253	Predictive accuracy = 82% on initial cohort, and 91% on independent cohort	No

*Table shows a summary of the different studies mentioned in this review*.

This limitation will prove to be challenging to overcome in neuro-ICU research as neuro-ICUs tend not to have large patient volumes, and the disease processes often presenting in the neuro-ICU are diverse (1). Software that easily collects and integrates waveform and imaging data with electronic medical records, i.e., Sickbay and CNS Monitor, could alleviate this issue, but their implementation so far has been sparse (49, 50).

Additionally, storing and sharing these datasets could prove challenging as well. Computational technology such as cloud-based storage systems have become abundantly available, allowing for easier storage and access to large datasets from around the world, though, one must be conscientious for the protection of private health information (51).

Finally, MLs are often seen as complicated to implement into studies; however, web-based platforms such as RapidMiner and Caffe deep learning framework can help those who are not experienced with ML coding by providing an automated ML-pipeline (52, 53). This increases the convenience of implementing MLs as time and skill-demanding codes can be streamlined for faster analysis.

## Future Directions and Conclusions

The neuro ICU is an optimal setting for ML application. MLs have been shown to efficiently analyze and condense the large, multi-dimensional data gathered from the neuro ICU to aid decision-making. In the future, the development of quantum processors, such as the Sycamore processor by Google, could improve the efficiencies of more complex MLs when analyzing extremely large datasets that are obtained at real time (54). For example, these processors could allow for MLs to continuously train itself and analyze data collected from the cloud, wearable devices, monitors, images, etc. to give real time feedback to ICU staff about their patients. Overall, it is an exciting time for ML-based applications, and all clinicians should become aware of the impact MLs will have on patient care. Specifically, due to the high data burden uniquely experienced by neuro ICU staff, neuro ICUs should begin to inquire about how MLs could improve their efficiency.

## Author Contributions

FC and RH contributed equally to the literature search and compilation of this article. PH, SA, SS, EK, CB, KJ, PL, and HN all contributed to different sections of the review. IL served as the senior author of the manuscript and supervised the compilation of sections from first-authors FC and RH and from co-authors. All authors contributed to the article and approved the submitted version.

## Conflict of Interest

KJ reported personal fees and other from Tempus Labs, other from Oova, Inc, personal fees from Thorne, outside the submitted work. The remaining authors declare that the research was conducted in the absence of any commercial or financial relationships that could be construed as a potential conflict of interest.
